# Oxidative Stress-Related Mechanisms in SARS-CoV-2 Infections

**DOI:** 10.1155/2022/5589089

**Published:** 2022-03-08

**Authors:** Joanna Wieczfinska, Paulina Kleniewska, Rafal Pawliczak

**Affiliations:** Department of Immunopathology, Medical Faculty, Medical University of Lodz, Lodz, Poland

## Abstract

The COVID-19 pandemic caused relatively high mortality in patients, especially in those with concomitant diseases (i.e., diabetes, hypertension, and chronic obstructive pulmonary disease (COPD)). In most of aforementioned comorbidities, the oxidative stress appears to be an important player in their pathogenesis. The direct cause of death in critically ill patients with COVID-19 is still far from being elucidated. Although some preliminary data suggests that the lung vasculature injury and the loss of the functioning part of pulmonary alveolar population are crucial, the precise mechanism is still unclear. On the other hand, at least two classes of medications used with some clinical benefits in COVID-19 treatment seem to have a major influence on ROS (reactive oxygen species) and RNS (reactive nitrogen species) production. However, oxidative stress is one of the important mechanisms in the antiviral immune response and innate immunity. Therefore, it would be of interest to summarize the data regarding the oxidative stress in severe COVID-19. In this review, we discuss the role of oxidative and antioxidant mechanisms in severe COVID-19 based on available studies. We also present the role of ROS and RNS in other viral infections in humans and in animal models. Although reactive oxygen and nitrogen species play an important role in the innate antiviral immune response, in some situations, they might have a deleterious effect, e.g., in some coronaviral infections. The understanding of the redox mechanisms in severe COVID-19 disease may have an impact on its treatment.

## 1. Introduction

Patients with pneumonia of unknown etiology had been diagnosed in mid-December 2019 in Wuhan (Hubei province, China). Later, the SARS-CoV-2 (severe acute respiratory syndrome) coronavirus started to spread all over the world, without any exemptions. As for today, more than 182 million of patients have been infected, and more than 3.9 million died due to COVID-19 [1], providing an estimate of the mortality rate at 3.3%. When compared to the seasonal flu, COVID-19 related mortality is at least 60 times higher. Seasonal flu outbreak annually causes the infection of 3 to 5 million people, both asymptomatic and symptomatic, with mortality rate not exceeding 0.05% [2]. Clinical course of COVID-19 may, in most cases, consist of three periods [3]. After a short incubation period lasting from 2 to 5 days, patients become symptomatic, with the loss of sense of taste and olfactory dysfunction, dry cough, fever exceeding 38°C, and dyspnoea. Other symptoms, including headache, fatigue, diarrhoea, and conjunctivitis, are less frequent. Additionally, most patients develop a bilateral interstitial pneumonia [4]. After 7-10 days, dyspnoea decreases in majority of patients, inflammatory changes in the lungs resolving to some extent, and the patients are free from the virus in most cases. In severe COVID-19, the pneumonia causes a rapid drop in arterial pO_2_ levels with the transcutaneous saturation measurement, usually below 60% when breathing ambient air. The progressive respiratory failure due to the loss of lung active surface of gas exchange and vascular abnormalities leads to the need of noninvasive ventilation support. In most severe cases, patients suffer from disseminated intravascular coagulation (DIC) or a septic shock and have to be sedated and undergo ventilation support [5].

Some experimental data available so far has suggested that the severe COVID-19 course might be related to the viral load during the SARS-CoV-2 exposure [6]. A recent study performed in 1145 patients suggested a significant independent association between viral load and mortality (with the hazard ratio of 1.07 [95% CI 1.03–1.11], *p* = 0•0014) implying that the 7% increase in mortality risk was present for each log transformed copy of viral RNA per mL of nasopharyngeal swab sample [7]. Another important factor, probably protecting from the development of severe COVID-19, is a normal to high level of serum vitamin D [8, 9]. Smoking cigarettes, however, may increase the risk of severe course of the disease, even in the absence of smoking-related disease [10–12]. The well-known and widely accepted hypothesis is that the male sex, hypertension, COPD, diabetes, or cancer may deeply influence the severity of the disease [13–16].

Today, it is not clear whether bronchial asthma may have any effect on the infection rate or the severity of COVID-19. Moreover, the question of how and why the viral pneumonia leads to DIC and septic shock with cytokine and bradykinin storms remains to be elucidated. ROS and RNS play an important role in the innate immune response, which is also directed against viruses [17]. In this review, we focus on the possible role of ROS and RNS in severe COVID-19 pathogenesis.

## 2. Antiviral Immune Response Mechanisms

**Figure 1:**
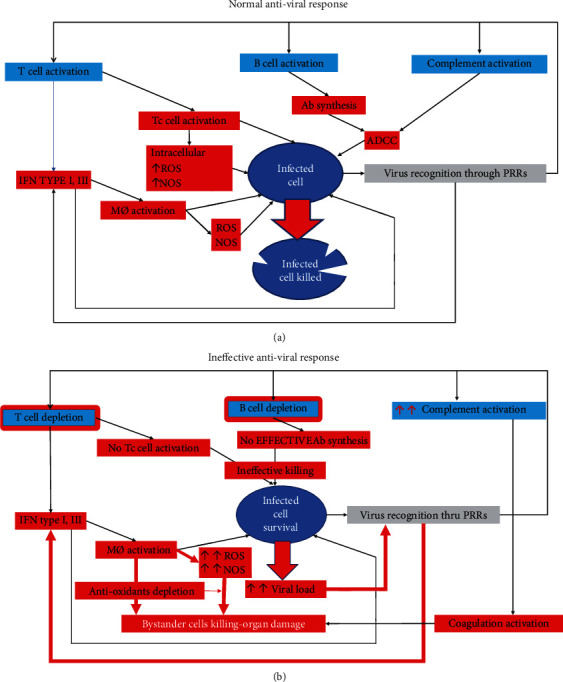
Normal (a) and ineffective (b) antiviral response. Under normal conditions, the presence of a virus activates various pathways leading to its killing by killing the infected cell: activation of complement, B and T lymphocytes, secretion of interferons, antibodies production, and macrophages activation, which result in an increase in ROS and NOS concentrations that help kill the infected cell. An ineffective antiviral response may occur when the responses to the presence of the virus are unnaturally enhanced, resulting in damage to surrounding tissues and as a consequence, organ damage. ADCC: Antibody-dependent cellular cytotoxicity.

The immune system has the potential to effectively control viral infections, and thus, it can limit their effect on the host organism. The processes of virus entry into the host cell, its replication, stimulation, and regulation of the antiviral immune response trigger a complex series of interactions between the virus and the host [18] (Figure 1(a)). There are two defense mechanisms: specific, acquired immunity and nonspecific, innate immunity. Nonspecific immunity is the first line of defense against infection and does not depend on prior contact with the pathogen. Mast cells, NK (natural killers), NKT (natural killer T cells), NHC (natural helper cells), natural lymphoid cells, granulocytes, macrophages, and monocytes are responsible for innate immunity. The pathophysiology of the extremely high pathogenicity of coronaviruses is not fully understood [19, 20]. It is worth noting that the immune system must develop a specific cytotoxic T cell (CTL) response. CTLs have the ability to recognize the viral-derived peptide on the surface of the infected cell, specifically in the MHC (the major histocompatibility complex) class I binding groove. Then, lymphocytes recognize the infected cell and destroy it by secreting cytolytic granules or activating programmed death in the cell through receptors such as FAS. In parallel with the development of the cellular response, a humoral immune response develops—associated with the activation of B cells and the subsequent release of specific antibodies. The helper T cells are at the center of the activation of adaptive immunity.

The lung epithelium is the largest surface that comes into contact with the environment. In the airways, viruses are detected by airway epithelial cells, mast cells, and cells of the mononuclear phagocyte system. The sensor cells are equipped with pattern recognition receptors such as Toll-like receptors (TLR). PAMPs (pathogen associated molecular patterns), derived from viruses, trigger a specific combination of PRRs (pattern recognition receptors) and adapter molecules, leading to the immune response adapted to the pathogen [21]. Coronavirus replication leads to, e.g., disruption of lysosomes, damage of mitochondria or/and imbalanced ion concentrations [22, 23]. As a consequence, pyroptosis occurs, which initiates the secretion of proinflammatory molecules of the interleukin-1 family [24, 25] (Figure 1(b)). Coronavirus SARS-CoV-2-induced cell death releases histones and a high-mobility group box 1, which are normally hidden from recognition by PRRs. Then, additional proinflammatory cytokines and chemokines are produced, e.g., IL-6, IP-10, MIP1*αβ* (macrophage inflammatory proteins-1*αβ*), and MCP1 (monocyte chemoattractant protein-1). Only in theory, detection of CoVs by pattern recognition receptors triggers an innate immune response that would be effective to limit viral replication. Interferons (IFN)-*α*, *β*, and type III are released to help control/eliminate viral infection. Their function is to remove the virus from infected cells by activating ISGs (IFN-stimulated genes) which exert direct antiviral effects, i.e., recruit antiviral immune effector cells. It has been observed that during zoonosis, the antiviral immune response can be detrimental to the body if the timing and target tissue of the immune response are inadequate [26].

The mechanism of innate immunity leads to inflammation, release of IFN-*αβ*, and activation of NK cells, which allows the suppression of local infection. Unfortunately, coronaviruses have developed strategies to protect themselves or their by-products from being recognized by the host [27]. In addition, viruses inhibit interferon induction and block IFN signaling. For example, SARS-CoV-1 (the coronavirus emerged in 2003, causing severe acute respiratory syndrome coronavirus) can effectively suppress interferon expression by nonstructural and structural proteins [28]. Coronaviruses circumvent the early phase of the innate immune response. Generalizing, the virus is recognized due to the stimulation of Toll-like receptors located on the epithelium and on dendritic cells, which are designed to inform B and T lymphocytes about the invasion of the pathogen. In the case of coronaviruses, these are Toll-like receptors 7 and TLR8 receptors that recognize viral RNA. Viral proteins are recognized by TLR2 and TLR4 receptors. During SARS-CoV-2 infection, the level of these receptors decreases, and their expression is lower in the elderly. SARS-CoV-2 infection is dangerous when a patient lacks specific antibodies and specific CTLs, because it can progress to severe pneumonia and ARDS [29].

## 3. The Role of ROS and RNS in Antiviral Response

The generation of ROS is one of the major mechanisms leading to infected cell death through apoptosis or necrosis, specifically during the very early stages of the immune response [30]. Both ROS and RNS also play an important role in signal transduction. Viral proteins or nucleic acids triggering the pattern recognition receptors lead to activating the interferon response through TIR-domain-containing adapter-inducing interferon (TRIF) and interferon regulatory factors (IRFs) as well to increasing in the inducible nitric oxide synthase (iNOS) expression and activity through the myeloid differentiation primary response-88 (MyD-88) adapter protein [31]. These processes lead to an increase in the RNS production. The RNS might inhibit viral proliferation in infected cells [32, 33]. Similarly, both PRRs and interferon type I pathways lead to an increase in ROS production from the xanthine oxidase, nitric oxide synthase, or the mitochondrial respiratory reactions. These processes have been crucial in the innate immune response to various viruses including human respiratory viruses (influenza viruses, HRSV (human respiratory syncytial virus), and rhinoviruses).

ROS are signaling molecules regulating a wide variety of physiological functions. ROS are a part of the mechanisms leading to the elimination of virus-infected cells and patient recovery. In some rare cases, specifically in the case of influenza infection, a severe course of the disease develops, leading to a severe adult respiratory distress syndrome (ARDS) with significant mortality [34]. Why ARDS is more frequent in some coronavirus infections (SARS, MERS (Middle East respiratory syndrome coronavirus), and SARS-CoV-2) remains unknown. Therefore, ROS and RNS might be at least one of the important diseases modifying pathways in severe COVID-19.

The effects caused by the reactive forms of oxygen and nitrogen might depend on the source of their origin. For instance, many RNA viruses activate endosomal NADPH (nicotinamide adenine dinucleotide phosphate hydrogen) oxidase *via* Toll-like receptor 7 mechanism, activated in turn by binding to single-stranded RNA [35, 36]. This is likely because these viruses, when attached to the cell, are built into the endosomes and their RNA can interact with TLR7; SARS-CoV-2 might activate Nox2 (NADPH oxidase 2) through TLR7 and that might have a negative impact on the defense mechanism against viruses. This is due to the fact that Nox2 activation is used by viruses in order to restrain immune reactions and develop the infection [36, 37].

**Figure 2:**
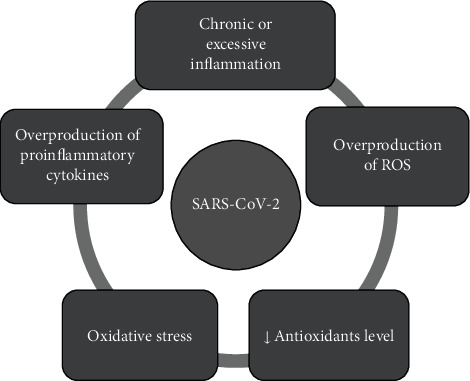
Molecular vicious circle of SARS-CoV-2 infection. Chronic or excessive inflammation damages tissues due to huge amounts of various toxic substances mainly ROS overproduced by cells of the immune system (neutrophils and macrophages). Activated phagocytes can also release prooxidant cytokines, e.g., TNF-*α* (tumor necrosis factor-alpha) and IL-1, which promote iron uptake by the reticuloendothelial system. The consequence of an uncontrolled inflammatory reaction is oxidative stress, which in turn, stimulates the inflammatory cells to further produce cytokines. Release of interleukins, e.g., 1*β*, 2, 6, 7, 12, 17, and TNF-*α* has been observed in COVID-19 [13], resulting in a vicious circle.

Overproduction of toxic ROS and excessive inflammation are harmful for tissues and may cause their damage [38–41]. As a result of an uncontrolled inflammatory response, oxidative stress (an imbalance between oxidants and antioxidants) arises, which in turn stimulates inflammatory cells to further produce cytokines and a “vicious circle” occurs (Figure 2).

**Figure 3:**
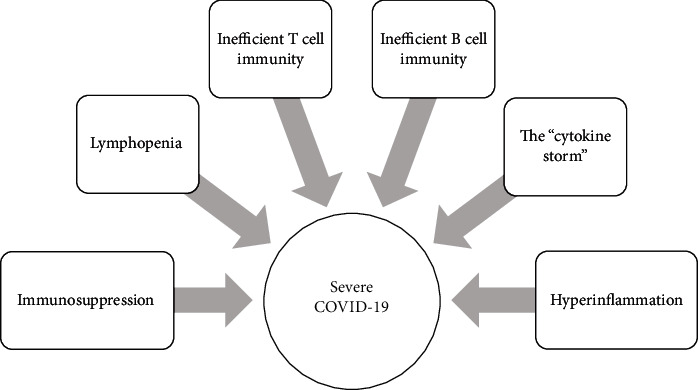
Immune changes characteristic of serve COVID-19. Deregulation of cytokines and influx of inflammatory cells can lead to lung infiltration and critical symptoms; a “cytokine storm” may lead to a dramatic disruption of the homeodynamics of the whole organism and, consequently, even death of the patient [24].

The characteristic features of the severe form of COVID-19 include, but are not limited to, severe lymphopenia, lung tissue damage, and a “cytokine storm” leading to acute respiratory distress and multiorgan failure. Despite a central role of mitochondria in ROS generation, many questions remain unanswered about their role during the “cytokine storm” and pathogenesis of infections with coronaviruses. Lymphopenia causes, among others, a defect in the regulation of antiviral immunity. The cytokine storm begins with the intense activation of cytokine-secreting cells with innate and acquired immune mechanisms [42] (Figure 3). It should be pointed out that in the case of a “cytokine storm”, neutrophil apoptosis does not occur. Patients have a huge amount of neutrophils that have undergone NETosis (NET-neutrophil extracellular traps). During NETosis, neutrophil extracellular trap is formed, and along with the “spilling out” of neutrophil DNA outside the cell, toxic enzymes are released, such as elastase, which damages lung tissue [43]. Moreover, microclots in the pulmonary circulation are formed. In the blood of COVID-19 patients, immune changes characteristic of viral infections were observed, i.e., increased levels of ASC-producing cells, activated CD4+ T cells and CD8+ T cells and IgM and IgG antibodies [44, 45]. Importantly, “cytokine storm” may occur, responsible for lung tissue damage during viral respiratory infections [46, 47]. Such sustained ROS production leads to the vicious circle that results in inflammatory damage but also hinders treatment of damage [48].

## 4. ROS and RNS Generation in SARS, MERS, and COVID-19

The high mortality rates of SARS-CoV-1, SARS-CoV-2, and MERS motivate scientists to study these infections in a variety of ways to find any effective therapeutic options. While numerous studies confirm a strong association between oxidative and nitrosative stress and severity of various viral infections (HCV (hepatitis C virus) [49], HBV (Hepatitis B virus) [50], and HRSV [51]), there is still limited clinical data showing such dependence in case of the SARS-CoV, SARS-CoV-2, and MERS infection—their severity or progression [52]. Previous research demonstrated that in SARS-CoV-infected human lung samples, explicit production of oxidized phospholipids followed by ROS generations was observed in the injured air spaces, pneumocytes, and alveolar macrophages [53]. Moreover, in macrophages, the oxidized phospholipids have been shown to modulate lung injury severity by TLR4-TRIF-TRAF6 expression and trigger cytokine production [22]. Lin et al. published a study showing that the ROS-activated NF-*κ*B (nuclear factor kappa-light-chain-enhancer of activated B cells) signal transduction pathway is induced by SARS-CoV-1 protease-3CLpro and therefore might be involved in the SARS-CoV infection development [54].

Angiotensin converting enzyme-2 (ACE2), known as the cell entry receptor of the SARS-CoV-2, is a multifunctional transmembrane protein. ACE2 plays a double-edged role in SARS-CoV-2 infection, and apart from being the cellular receptor for SARS-CoV-2 spike proteins, it is the critical molecule in combating inflammatory and oxidative damage of tissues by COVID-19. This enzyme decreases angiotensin II which is stimulant of NADPH oxidase. In addition, the product of ACE2 enzymatic activity, angiotensin 1-7, has a strong antioxidant effect [55, 56].

The virus binding to ACE2 receptor initiates its entry to the cell, and after attachment and virion-membrane fusion, ACE2 expression is downregulated [57, 58]. The viral protein Spike interaction with ACE2 leads to an excessive production of angiotensin II (Ang II) and activation of NADPH oxidase which subsequently results in enhancing oxidative stress mechanisms (in contrast to what happens during other viral infections) but also releasing inflammatory molecules [59]. In the course of SARS-CoV-2 infection, angiontensin II availability is increased through the high affinity and resulting binding the virus to ACE2 [60]. ACE2 in SARS-CoV-infected cells has been shown to be also involved in postinfection regulation, including immune response, viral genome replication, and cytokine secretion [61]. A previous study demonstrated that overexpression of ACE2 prevents Ang II-induced Nox2 expression and ROS generation in endothelium [62]. In healthy individuals, ACE2 supports lung homeostasis *via* the production of angiotensin 1–7 and controls inflammation and blood pressure. However, ACE2 downregulation may prevent SARS-CoV-2 host cell interaction in chronic respiratory conditions [63]. ACE2 is expressed in a variety of cells. It has been shown that many factors can influence the changes in ACE2 expression and the progression of COVID-19, including gender and age [64].

The severity of coronavirus infections is generally age related [65], which might be attributed to a disruption in the redox balance, i.e., accumulated oxidative damage and a deteriorated antioxidative defense system followed by increased reactive oxygen species [66]. As a consequence, induction of proinflammatory cytokine expression occurs (such as TNF-*α*, interleukin (IL) 6, IL-8, and IL-1*β*), *via* redox-sensitive transcription factors, e.g., NF-*κ*B [67, 68]. Previous genomic analyses of SARS-CoV-1 on aged macaques demonstrated that old subjects presented stronger host response to virus and more severe infection pathology than young ones; this was associated with a reduced expression of type I interferon and an increase in the differential expression of inflammatory genes related to NF-*κ*B [66].

Recent study demonstrated that patients suffering from severe COVID-19 disease, requiring intensive care unit treatment, presented higher levels of Nox2 activation, and thus, Nox2 seems to be a pivotal agent in COVID-19 aggravation [37]. However, Li et al. published data suggesting that the SARS-CoV nonstructural protein nsp10 might impair the redox system in the mitochondria, another ROS source, by a loss in the cellular inner mitochondrial membrane potential. This effect probably enhanced the cytopathic effect of SARS-CoV-1 [69]. Interestingly, it has been shown recently that coronaviruses, thanks to the protein nsp10 in combination with nsp16, can methylate the 5 ′ends of their mRNAs, thus resembling the host mRNA and protecting them from the innate immune response [70].

**Figure 4:**
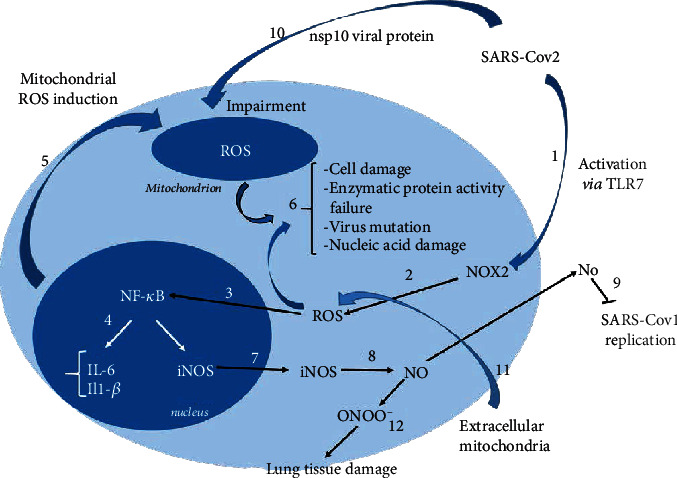
Action of SARS-CoV-2 in proposed mechanisms in the context of reactive oxygen and nitrogen species. SARS-Cov-2 may affect the induction of reactive oxygen species by inducing both of their sources—NADPH oxidase and mitochondria. The increase in Nox2 activity in COVID-19 patients may be related to the activation of this enzyme by TLR7 (1), as is the case with other RNA viruses. Activated NADPH oxidase is responsible for the production of ROS (2), which are related to the activation of NF-*κ*B (3). The activity of this transcription factor results in the expression of proinflammatory cytokines like IL-6 and-1*β* (4), which in turn can induce the production of mitochondrial ROS (5). On the other hand, ROS, if produced in excess, regardless of the source, may cause cell damage, enzymatic protein activity failure, virus mutation, and nucleic acid damage (6). NF-*κ*B, activated by ROS, has been proved to induce the expression of iNOS (7). The enzyme, responsible for the production of nitric oxide (8), has been shown to inhibit SARS-Cov virus replication (the coronavirus causing severe acute respiratory syndrome coronavirus, emerged in 2003), (9). Based on the analogy and similarity between SARS-Cov and SARS-Cov-2, it may be assumed that the nonstructural protein nsp10 causes mitochondrial impairment (10). Additionally, extracellular mitochondria, which are also ROS source, are able to provoke the immune response, regulate cell-to-cell communication and danger sensing (11). Peroxynitrite is formed by the reaction of nitrite (NO^•^) and hydrogen peroxide (12), and it has been proved to damage lung tissue and thus playing an important role in lung destruction in viral infections.

Moreover, inflammatory cytokines-TNF-*α* and IL-6, which may initiate mitochondrial ROS production and are associated with ATP production, were found in COVID-19 serum (Figure 4) [71, 72]. In fact, Saleh et al. proposed recently a hypothesis that, apart from the intracellular mitochondria failure that plays a key role in COVID-19 disease, the extracellular mitochondria are important mediators [73–76]. They provoke the immune response, regulate cell-to-cell communication, and danger sensing [77]. According to the authors, this complex interplay between platelet mitochondrial dysfunction, oxidative stress, and mitophagy would provide useful therapeutic strategies [73]. The excess of ROS can oxidize biomolecules (lipids, proteins, and DNA) or it can structurally modify proteins and genes to trigger signaling cascades that can lead to an inflammatory response. SARS-CoV-2 infection intensifies the already existing oxidative stress in patients of older age with comorbidities, e.g., diabetes, hypertension, and cardiovascular diseases and that is one of the possible explanations for the severity of COVID-19 in these categories of patients [52, 78].

**Figure 5:**
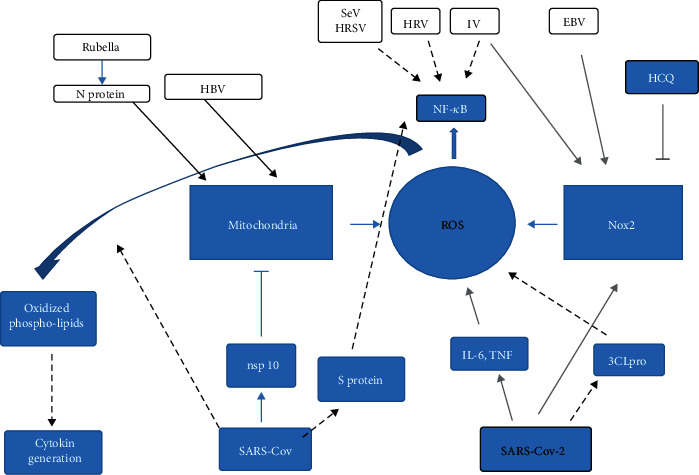
Chosen features of ROS/RNS metabolism during the COVID-19 in comparison to other viral infections. Nox 2, as the reactive oxygen species source, has been reported to be increased in COVID-19 patients [46], and, hydroxychloroquine (HCQ), the antimalarial drug, was demonstrated to inhibit Nox2 activity [48]. Nox increased activity is also a common feature of EBV and influenza virus infection, and Nox2 inhibition in IV infection lowers viral burden. On the basis of feedback, viruses (HRSV, HRV, and SeV), cause ROS induction via Nox2 [45, 74–76, 86]. In serum of COVD-19 patients, inflammatory cytokines (TNF-*α* and IL-6) were increased, possibly taking part in the initiation of mitochondrial ROS production [52, 53] (thick grey lines). Coronavirus SARS-CoV-2 protease–3CLpro has been shown to act through mitochondrial ROS, inducing NF-*κ*B signal transduction pathway [20]. This pathway is closely related to many other viral infections, e.g., SeV, HRV, IV, and SARS-Cov (via S protein) [74–76, 89]. The latter virus, by induction of ROS, and subsequent generation of oxidized phospholipids, may not only modulate the severity of acute lung injury but also directly induce inflammatory cytokine production in macrophages [19] (black, dotted line). Mitochondria are proved to be important ROS source in rubella and HBV infection [79, 80], as they induce ROS production, either directly, or via viral N protein (in case of rubella, black thick lines).

The above mentioned Nox2 is a multisubunit protein, and its activation requires translocation of the cytosolic subunits—p47phox, p67phox, and Rac to the NOX/p22phox membrane complex [79]. Superoxide produced by Nox2 is implicated in influenza-mediated lung pathology [80]. Tang et al. published studies suggesting that endosomes are the main site of ROS production under the influenza virus infection [46]. In addition, the authors indicated that ROS generation might be triggered by influenza virus in endosome *via* four different ways, one of which is TLR7 activation through the single-stranded RNA and protein kinase C activation. This results in phosphorylation of p47phox and by the assembly of the Nox2 oxidase complex at the endosomal membrane. The importance of Nox2 in influenza A infection was confirmed by literature, showing that in the absence of Nox2, influenza A virus results in lower viral burden and consequently results in significantly less lung injury, suggesting that ROS generated by Nox2 promotes rather than inhibits viral infection [80–83] (Figure 5.).

As mentioned earlier, apart from Nox2, also Nox1, Nox 4, and Duox2 might play a role in the ROS formation of viral infections [84–86]. Nox1 was shown to critically inhibit the early burst of proinflammatory cytokine expression in the lung and subsequently—oxidative stress followed by influenza A virus infection [85]. Nox1 oxidase has been proved to suppress early proinflammatory cytokine expression burst. Taking into consideration that ROS contribute to dysfunction and injury of the lung during influenza virus infection, this role of Nox1 seems surprising [85]. On the contrary, the study of Hofstetter et al. demonstrated that Nox1 presents activity promoting mortality during the peak of influenza infection, through restrain of the early phase of the adaptive immune response [87].

One of the key mediators of cytokines/chemokines induction is NF-*κ*B. The pathway of this transcription factor is directly activated by ROS and by certain proinflammatory cytokines, such as TNF-*α* and IL-1*β*. A wide spectrum of cytokines and chemokines may be expressed as a consequence of NF-*κ*B action, including IL-1*β*, IL-6, and IL-8, produced by most viruses (e.g., influenza virus, HBV, HIV (human immunodeficiency virus), EBV, SARS-CoV-2, and MERS); moreover, many respiratory viruses induce NF-*κ*B signaling both *in vitro* and *in vivo* in a ROS-dependent fashion [88–92]. During viral infections, NF-*κ*B binds to distinct sites of the iNOS promoter, causing iNOS enhanced expression. NO overproduction is predominantly caused by iNOS, which might be expressed, e.g., by inflammatory phagocytic cells [93–95]. Reactive nitrogen species play an important role in viral infections, in fact, some viruses, e.g., HCV, HRSV, or HIV, might upregulate the expression of iNOS [96–98]. On the other hand, IL-10, produced by many viruses (e.g., EBCV, HBV, HIV, SARS-CoV-2, and MERS), indirectly inhibits iNOS by inducing arginase, which reduces the availability of L-arginine, the substrate of iNOS [99, 100]. A previous study shows that HRSV directly upregulated iNOS in human type 2 alveolar epithelial cells, suggesting that the expression increase might be associated with interferon regulatory factor 1, instead of cytokines [101]. The regulatory factor mentioned has been involved in iNOS expression activation together with NF-*κ*B and double-stranded RNA-activated protein kinase, as shown previously in influenza virus infection [102].

## 5. Molecular Mechanisms of ROS and RNS Generation in Response to Viruses

Reactive oxygen and nitrogen radicals are generated, among others, in viral infections. In conditions of infection, ROS and RNS production might be activated either by viral components or by cytokines, in response to the pathogen. Apart from the influenza virus, several viruses are associated with ROS generation. Epstein-Barr virus (EBV) may induce generation of ROS by NADPH oxidase in B lymphocytes through upregulating Nox2 [103]. In turn, Nox4 is activated by core protein of HCV, but this oxidase initiates mitochondrial ROS production, showing that this virus induces other proteins to trigger ROS generation [104, 105]. Mitochondrial ROS generation pathway is activated by viruses in various ways—the rubella virus produces N protein which induces the production of ROS by increasing the activity of mitochondrial respiratory chain [106]. Yuan et al. demonstrated that hepatitis B virus increases mitochondrial ROS generation resulting in the elevated IL-6 expression [107].

Apart from mitochondrial source, ROS are produced by enzymatic activity of NADPH oxidases in a highly regulated manner and play roles in both physiology and disease [108]. Out of seven NADPH oxidase homologs, four are implicated in ROS generation under viral infections: Nox1, Nox2, Nox4, and Duox2, but the primary source of inflammatory cell ROS is the Nox2 oxidase enzyme [36, 109, 110]. Although Nox2 is a phagocytic enzyme playing a role in killing bacteria and fungi, it is also known for contributing to virus-induced ROS production during viral infections, e.g., with IV (Influenza virus) [111], HRSV [91], HRV (human rhinovirus) [112], SARS-CoV-2 [37], and SeV (sendai virus) [91, 92].

In severe COVID-19, the major cytokines generated as part of immune response are IL-1*β*, IL-2, IL-6, and TNF. Also, IFN-*ϒ* seems to play an important role in antiviral response, although the data may suggest some defective interferon synthesis and release in severe patients infected with SARS-CoV-2. IL-1*β* is a well-known ROS and RNS generation activator [113]. Similarly, IL-2 stimulates RNS to generate nitrogen radicals [114]. Interleukin-6 activates human neutrophils and monocytes increasing the generation of free oxygen radicals [115]. Similarly, IFN-*ϒ* and TNF stimulate the generation of RNS in human [114]. On the other hand, free oxygen radicals may increase IL-6 production and free nitrogen radicals are responsible, at least in part for its synthesis [116, 117]. Moreover, high levels of IL-6 are associated with the higher mortality rate in ICU- (intensive care unit-) treated COVID-19 patients [118, 119].

The effects of the antiviral potential of nitric oxide (NO) against SARS coronavirus have been described in Vero E6 cells and revealed, that NO donor, S-nitroso-N-acetylpenicillamine inhibited the replication cycle of SARS-CoV in a dose-dependent manner [67]. In patients with SARS, NO was associated with oxygenation amelioration. Moreover, endogenous but also exogenous NO inhibited SARS-CoV viral replication [120–122]. NO reacts with superoxide radicals yielding peroxynitrite, and both peroxynitrite and NO are toxic to mitochondria.

Apart from iNOS induction in response to viruses and viral components, interferon gamma has been reported as a major cytokine to induce iNOS and NO overproduction in the pathogenesis of virus infection [123, 124]. This cytokine is associated to Th1 cell response, as it is acknowledged that antiviral adaptive response is Th1 type [125]. Nevertheless, some viruses (such as influenza virus and HSV) might inhibit Th1 response through downregulation of interferons production. This type of immune response manipulation may prominently influence the consequence of the infection [126, 127]. Moreover, produced in excess during viral infection, reactive nitrogen species, are likely to influence mutagenesis in the virus [128].

## 6. The Possible Therapeutic Approach Related to Oxidative Stress Tampering in COVID-19

Several strategies for treating the SARS-CoV-2 infection are currently under consideration. Scientists and doctors have developed therapies based on the use of interferons, antibodies, inhibitors of viral/host proteases, and host-directed therapies. To date, no clinically effective antiviral therapy against SARS-CoV-2 has been confirmed; therefore, patients receive mainly supportive treatment which is often supplemented with various drug combinations. Many authors have documented elevated chemokines and interleukins levels in COVID-19 patients, so future efforts should focus inter alia on drugs that can be rapidly deployed and have immunomodulatory properties [129–132]. The use of interleukin 1 receptor antagonist in nine patients with moderate to severe COVID-19 pneumonia was effective in improving clinical and biological indices [133]. IL-1 receptor blocker reduced the need for invasive mechanical ventilation in the intensive care unit as well as mortality in patients with severe COVID-19 [134]. Shakoory et al. [135] in their randomized controlled trial confirmed that the inhibition of IL-1 receptor significantly decreased mortality in sepsis patients with features of macrophage activation syndrome. Patients who received IL-6 receptor antagonists had a marked reduction in pyrexia within days after treatment and a reduction in oxygen demand [136]. In the TESEO (the tocilizumab in patients with severe COVID-19 pneumonia) study, the use of a recombinant humanized antihuman IL-6 receptor monoclonal antibody (i.v. or s.c.) was associated with a reduced risk of mechanical ventilation and death [137]. Another IL-6 receptor blocker was effective only in critically ill COVID-19 patients requiring mechanical ventilation or high-flow oxygenation or requiring intensive care treatment [138]. Recent studies have highlighted the role of optimal nutritional status in boosting the immune system, focusing on the most important ingredients that reduce inflammation and oxidative stress parameters [139]. Interestingly, hydroxychloroquine (HCQ), the antimalarial drug, used to treat COVID-19, has been recently demonstrated to inhibit Nox2 activity through the ability to alkalize endosomes and therefore impedes antiphospholipd antibody activity (aPL) [35, 140]. The aPL, as a proinflammatory factor, has been proved to act *via* the pathway in which NADPH oxidase takes part [141]. There are many mechanisms for neutralizing free radicals, e. g., glutathione which is capable of affecting viral replication; the glutathione peroxidase/reductase enzyme system that allows reduced glutathione to bind to free radicals to produce oxidized glutathione, which is then regenerated to GSH; peroxyredoxin system that neutralizes lipid peroxidation; superoxide dismutase neutralizing superoxide anion; catalase eliminating hydrogen peroxide; carotenoids and polyphenols with scavenging effects; vitamins E and C; and finally, zinc and selenium, which have antioxidant properties as cofactors of antioxidant enzymes [142]. Providing substances that strengthen the antioxidant system will reduce the level of oxidative stress parameters during infection. Moreover, the use of molecular techniques to target antioxidants to organs of interest is an approach that might enhance the effectiveness of the antioxidant and circumvent toxicity [143].

Resveratrol is a wide studied antioxidative agent, which plays a role in mitochondria-derived ROS [144] but also down regulates the expression and activity of the NADPH oxidase [145]. In the case of MERS-CoV, resveratrol appeared to inhibit MERS-CoV infection. Moreover, the authors of a recently published study point out that as MERS-CoV infection leads to inflammatory cytokines production, resveratrol, via hindering NF-*κ*B pathway, may reduce the inflammation [146–148]. They also found that the expression of the nucleocapsid (N), which is essential for MERS-CoV replication, was decreased after resveratrol treatment [61]. MERS-CoV next to SARS-CoV-1 and SARS-CoV-2 has been demonstrated to depend on TMPRSS2 (transmembrane serine protease 2) which plays an important role during the virus entry to the cell. Presumably, TMPRSS2 might regulate mitochondrial function [149–151].

Recently, many others antioxidants have been tested for the highly conserved SARS-CoV-2 main protease using molecular docking. Of all the compounds that were investigated, the lowest predicted IC50 value was observed for taxifolin. Moreover, taxifolin along with other compounds such as eriodictyol did not show any toxicity against the toxicity parameters used in the experiment [152]. This flavonoid was found to be a powerful antiradical and antioxidant activities in different *in vitro* bioassays when compared with standard antioxidant compounds [153]. This compound inhibits NF-*κ*B pathway and downregulates STAT3 of the JAK/STAT pathway [154]. Thus, taxifolin could be a potential inhibitor against Mpro but further *in vivo* studies are needed [155]. Another analyzes also point to the natural compounds, taxifolin and rhamnetin, as potential inhibitors of Mpro [156]. Rutin (a polyphenolic flavonoid) may able a potential inhibitor as it is able to form several hydrogen bonds and *σ*-*π* stacking interactions with various amino acids of Mpro in anchoring and blocking the substrate into the active pocket of the catalytic center [157]. *In vivo* and in silico studies have demonstrated that silymarin and its derivative silybin (a flavonoid from the group of flavonolignans) are able to inhibit SARS-CoV-2 main protease [158]. Another authors found luteolin to be effective in blocking the S2 protein of SARS-CoV [159]. It is already known that the SARS-CoV and SARS-CoV-2 S proteins share about 76% amino acid similarity [142]. Several other herbal compounds like quercetin, naringenin, kaempferol, allicin, demethoxycurcumin, catechin, apigenin-7-glucoside, oleuropein, curcumin, zingerol or gingerol have been also investigated [57].

The approach of using antioxidants both to reduce viral replication and to reduce viral-induced oxidative damage may prove to be particularly useful for those viruses, which have thus far eluded attempts at antiviral therapies.

## 7. Conclusion

In conclusion, the literature demonstrates an important role of reactive oxygen and nitrogen species during SARS-CoV-2 infections, associated with a weakened antioxidant defense. Nevertheless, it must be noted that some of the understanding, background, and supporting data presented in the current review come from the experience with other human coronaviruses or viruses, such as RSV/HBV/HCV, and may not necessarily be known to be appropriate with respect to SARS-CoV-2.

The oxidative stress mechanism coupled with innate immunity activates transcription factors, such as NF-*κ*B, which results in an exacerbated proinflammatory host response. The importance of ROS and RNS is also connected with the fact that this virus is especially dangerous for the elderly, and their deteriorated antioxidative/nitrosative defense system affected by increased reactive oxygen and nitrogen species. Moreover, only treatments diminishing the ROS and RNS production such as dexamethasone and tocilizumab deliver substantial benefits to severe COVID-19 patients. Therefore, there is a strong need to deeply investigate this issue, as it would be of interest to use the antioxidants as potential therapeutic tools.

## Data Availability

The data supporting this review article are from previously reported studies and datasets, which have been cited. The processed data are available from the corresponding authors of upon request.

## Abbreviations

ACE2: Angiotensin-converting enzyme 2
ADCC: Antibody dependent cellular cytotoxicity
aPL: Antiphospholipd antibodies
ARDS: Acute respiratory distress syndrome
CTL: Cytotoxic T leukocytes
DIC: Disseminated intravascular coagulation
EBV: Epstein-Barr virus
HBV: Hepatitis B virus
HCV: Hepatitis C virus
HCQ: Hydroxychloroquine
HIV: Human immunodeficiency virus
HRSV: Human respiratory syncytial virus
HRV: Human rhinovirus
ICU: Intensive care unit
IFN: Interferon
IL: Interleukin
iNOS: Inducible nitric oxide synthase
IRFs: Interferon regulatory factors
ISGs: IFN-stimulated genes
IV: Influenza virus
MCP-1: Monocyte chemoattractant protein-1
MERS: Middle East respiratory syndrome coronavirus
MHC: Major histocompatibility complex
MIP1*αβ*: Macrophage inflammatory proteins-1*αβ*
MyD-88: Myeloid differentiation primary response-88
NADPH: Nicotinamide adenine dinucleotide phosphate hydrogen
NET: Neutrophil extracellular traps
NF-*κ*B: Nuclear factor kappa-light-chain-enhancer of activated B cells
NHC: Natural helper cells
NK: Natural killers
NKT: Natural killer T cells
NOX2: NADPH oxidase 2
PAMPs: Pathogen associated molecular patterns
PRR: Pattern recognition receptors
SARS: Severe acute respiratory syndrome
SeV: Sendai virus
TLR: Toll-like receptors
TMPRSS2: Transmembrane serine protease 2
TNF-*α*: Tumor necrosis factor alpha
TRAF6: TNF receptor associated factor 6
TRIF: TIR-domain-containing adapter-inducing interferon-*β*.
